# Chaperone-mediated autophagy, heat shock protein 70, and serotonin: novel targets of beta-hydroxybutyrate in HFFD/LPS-induced sporadic Alzheimer’s disease model

**DOI:** 10.1007/s10787-025-01754-6

**Published:** 2025-05-04

**Authors:** Reem A. Mohamed, Dalaal M. Abdallah, Hanan S. El-Abhar

**Affiliations:** 1 Department of Pharmacology and Toxicology, Faculty of Pharmacy, October University for Modern Science and Arts (MSA), Cairo, 12566 Egypt; 2 Department of Pharmacology and Toxicology, Faculty of Pharmacy, Cairo University, Cairo, Egypt

**Keywords:** Alzheimer disease, Chaperone-mediated autophagy, Hsp70, NLRP3, β-hydroxybutyrate, Serotonin

## Abstract

Sporadic Alzheimer’s disease (AD), which accounts for the majority of cases, is sturdily influenced by lifestyle factors such as dietary habits, obesity, and diabetes, leading to its classification as Type 3 diabetes. To model this pathological link, our AD-like model was developed by feeding Wistar male rats a high-fat diet with fructose in drinking water (HFFD) for 8 weeks, followed by a single dose of lipopolysaccharide (LPS). This group was compared with a normal control group fed a standard diet and a β-hydroxybutyrate (BHB)-treated group (125 mg/kg, p.o.), administered starting 3 h after LPS and continuing for 1 week. The results demonstrate that BHB treatment illuminated cognitive gains, as indicated by the Y-maze, Morris water maze, and novel object recognition tests. In addition, it preserved hippocampal cytoarchitecture, reduced neurodegeneration, and attenuated amyloid plaques and phosphorylated Tau deposition. Cellularly, BHB restored critical molecular mechanisms, including increased lysosomal-associated membrane protein 2A (LAMP2A) hippocampal content as the main marker of chaperone-mediated autophagy (CMA), along with the chaperon protein Hsp70. Moreover, BHB alleviated neuroinflammation by inhibiting the nucleotide-binding domain, leucine-rich–containing family, and pyrin domain–containing-3 (NLRP3) inflammasome activation alongside the downstream targets cleaved caspase-1 and IL-1β/IL-18 cytokines. BHB also reduced pyroptotic markers, caspase-11 and gasdermin-N, and microglia-induced inflammation as it shifted microglial polarization toward the neuroprotective M2 phenotype. Finally, BHB normalized hippocampal neurotransmitter levels of the inhibited acetylcholine and serotonin. These findings support BHB as a promising, multifaceted treatment for AD, highlighting the roles of CMA, Hsp70, and 5-HT in slowing disease progression and improving cognitive function.

**Graphical abstract:**

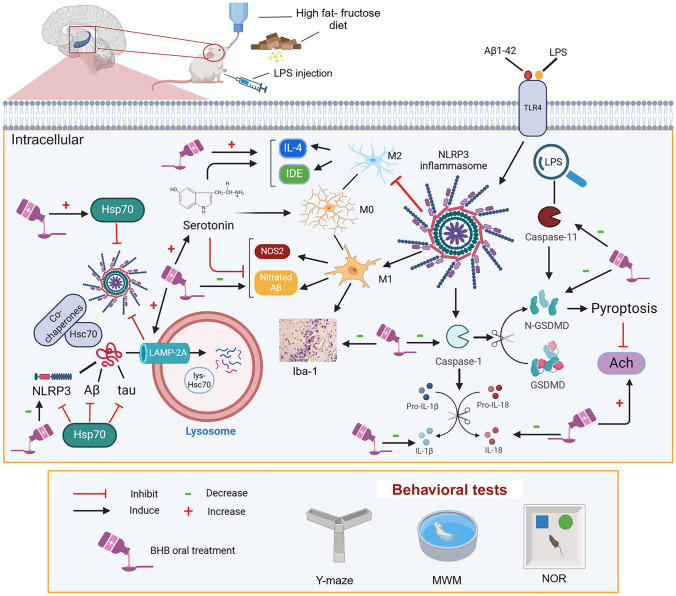

**Supplementary Information:**

The online version contains supplementary material available at 10.1007/s10787-025-01754-6.

## Introduction

A sedentary lifestyle is a dynamic influence on obesity and diabetes, conditions that jeopardize mental health and may predispose individuals to Alzheimer’s disease (AD) termed as Type 3 diabetes (Anfal and Hayder 2022). In AD, glucose metabolism and glucose transporter-1 (GLUT1) function are impaired, leading to reduced glucose uptake with insulin resistance (IR) progression, which contributes to cognitive deficits (De la Monte 2017; Rubio et al. 2025). Since the new millennium, the crosstalk between AD and diabetes has been recognized, as both conditions share risk factors such as high cholesterol, neurodegeneration, β-amyloid (Aβ) accumulation, oxidative stress, inflammation, metabolic dysfunction, and apoptosis (Stanciu et al. 2020). Moreover, insulin dysfunction plays a crucial role in neurological impairment, affecting acetylcholine (Ach) synthesis and Aβ clearance (Stanciu et al. 2020), with Aβ aggregation forming amyloid plaques, a key hallmark of AD.

Damaged, misfolded, or aggregated proteins are fragmented centrally by two primary protein degradation pathways, one of which is the autophagy flux pathway (Xilouri and Stefanis 2015). The function of chaperone-mediated autophagy (CMA) represents a selective process crucial for preserving protein quality and proteome steadiness by targeting the KFERQ motif-containing proteins (Kaushik and Cuervo 2018). This mainly relies on the charge of the residues in the sequence rather than the exact amino acid composition (Dice 1990). One of the heat shock proteins family, known as heat shock cognate (Hsc) 70, detects this sequence to bind and unfold target proteins after complexing with co-chaperones, conveying them to the lysosomal membrane (Dice 2007). There, a receptor known as lysosomal-associated membrane protein 2A (LAMP2A) facilitates their entrance into the lysosomal lumen to be recycled after being degraded (Bejarano and Cuervo 2010). CMA activity can be influenced by various factors, decreasing with aging (Dice 2007) and high-fat diet consumption in the liver (Portovedo et al. 2020 hypothalamus; Rodriguez-Navarro et al. 2012 Liver) while activated by calorie restriction (Jafari et al. 2024).

Heat shock protein (Hsp)70 is another chaperone protein that serves to ensure proper protein functioning by helping them fold properly, assists their subcellular transport, and governs their incorporation into complexes and their latter dissociation, and finally the degradation of defective, distorted, or amassed proteins (Mayer and Bukau 2005). Therefore, Hsp70 has a broad impact on protein balance by regulating protein surveillance and recycling under any settings (Meimaridou et al. 2009). This protein was also reported to be induced under stress conditions (Evans et al. 2010).

In sporadic AD human brains, extracellular lipopolysaccharide (LPS) and aggregated Aβ1-42 co-localize (Zhan et al., 2016) and activate microglia by interacting with toll-like receptor (TLR) 4, which primes nucleotide-binding domain, leucine-rich–containing family, and pyrin domain–containing-3 (NLRP3), and stimulate nuclear factor-kappa B (NF-κB), thereby promoting neuroinflammation. This activation facilitates inflammasome formation, where NLRP3 assembles with apoptosis-associated speck-like protein (ASC) and pro-caspase-1, a complex that cleaves caspase-1 causing the activation of interleukins (IL)-1β and IL-18, ultimately triggering pyroptosis (Ghoneim et al. 2020; Jia et al. 2024). The unconventional signal, however, involves caspase-11 activation via LPS (Kayagaki et al. 2011; Jorgensen and Miao 2015), with both pathways leading to gasdermin D (GSDMD) cleavage and pyroptotic cell death (Ye et al. 2020).

Another contributor to neuroinflammation is the activation of microglia, which shift toward the M1 phenotype pro-inflammatory while the M2 profile is reduced. Activated M1 microglia enhance nitric oxide synthase 2 (NOS2) activity, leading to Aβ nitration, which increases its aggregation and resistance to degradation by proteases like insulin-degrading enzyme (IDE). In contrast, M2 phenotype markers, including IL-4 and IDE, are diminished during neuroinflammation (Heneka et al. 2013). The reduction in IDE during neuroinflammation might promote the aggregation of nitrated Aβ.

Recently, the ketogenic diet (KD) appeared as a compelling supportive treatment for a range of disorders including obesity (Paoli 2014), diabetes (Dashti et al. 2021), and even certain cancers (Lane et al. 2021). Lately, its potential has extended to neurological diseases driven by chronic neuroinflammation, including AD (Broom et al. 2019) (Rubio et al. 2025). Centrally, KD was reported to reduce inflammatory cytokines, oxidative stress, excitatory neurotransmitters, and maintain mitochondrial function (Rubio et al. 2025). The metabolism of KD results in the production of beta-hydroxybutyrate (BHB) among other ketone bodies to act as back-up fuel for the brain during states of energy deficit or decreased glucose utilization (Cunnane et al. 2011; Cunnane et al. 2016).

Research on AD has expanded with new pathophysiological links discovered annually. With about 50 million people affected worldwide (Li et al. 2022), the increasing prevalence emphasizes the need for continued research. Although BHB has been lately acknowledged as a candidate in AD research owing to its neuroprotective potential (Jang et al. 2023), further molecular mechanisms remain to be explored. Thus, our goal is to scrutinize the effect of BHB in a high fat-fructose (HFFD)/LPS-induced sporadic AD model, focusing on its impact on CMA, Hsp70, NLRP3 inflammasome, and microglia activation to modulate Aβ and Ach/serotonin (5-HT) neurotransmitters.

## Materials and methods

### Animals

A total of 27 male Wistar rats aged 2 months and weighing 90–100 g were used in the experiment. They were purchased from the animal production facility of the National Research Center (Egypt) and housed under controlled environment at the MSA University with free access to water and chow. Experimental intervention started after 1 week to ensure animal’s acclimatization. The work was ethically sanctioned by the MSA University Ethics Committee (approval no. PH1/REC1/2023PD) and followed the recommendations in the Guide for the Care and Use of Laboratory Animals of the National Institutes of Health and abided by ARRIVE guidelines.

### Drugs/chemicals used

LPS (O55:B5) and BHB were purchased from Sigma-Aldrich Co. (MO, USA), whereas fructose (Unifructose^®^) was obtained from UNIPHARMA Co. in Egypt.

### HFFD/LPS-induced AD-like model

Sporadic AD-like model was induced according to a previous protocol performed in our laboratory (Mohamed et al. 2021). Briefly, animals (*n* = 18) were permitted to eat a high-fat diet (HFD) and to drink 20% fructose in drinking water for 8 weeks after which the animals developed a state resembling metabolic syndrome. In the serum of fasting animals, the levels of glucose, insulin, triglycerides (TGs), and total cholesterol (TC) were measured to characterize the occurrence of this syndrome. Homeostasis Model Assessment of IR (HOMA-IR) (Matthews et al. 1985) was calculated according to the following formula to assure insulin resistance (IR).

$${\text{HOMA}} - {\text{IR}} = \frac{{\left[ {\frac{{\text{fasting glucose}}}{18} \times {\text{fasting insulin }}} \right]}}{22.5}$$

Afterward, rats with IR were administered 2 mg/kg LPS intraperitoneally once after being dissolved in 0.9% saline, whereas the control group (nine rats) were allowed to intake normal-fat diet (NFD) and received saline injection.

### Research framework

**Fig. 1:**
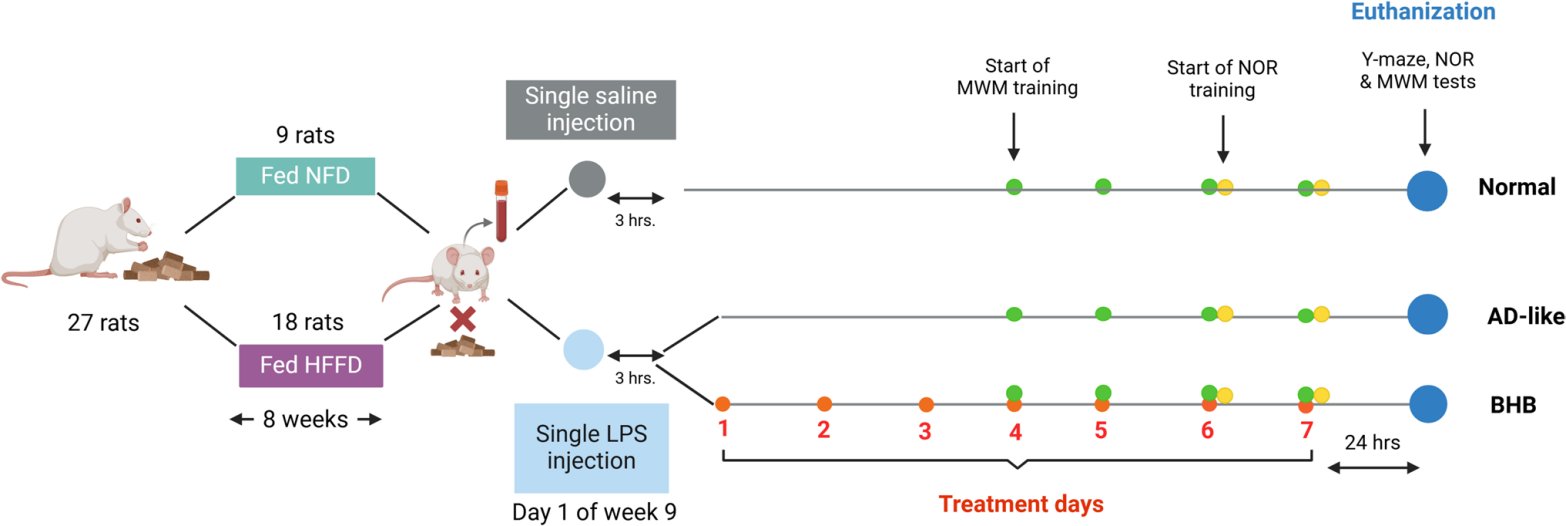
Experimental timeline. Rats fed HFFD for 8 weeks were injected with a single dose of LPS (2 mg/kg, i.p), but those fed NFD received a single saline injection. The untreated HFFD/LPS group are the AD-like model (Group 2), whereas the model-treated animals received BHB (125 mg/kg, p.o) for 7 days starting 3 h after LPS injection (Group 3). MWM training started from days 4–7, and the probe test was done on day 8. The NOR test lasted from day 6 to day 8, and the Y-maze test was performed on day 8 and rats were euthanized thereafter. AD: Alzheimer’s disease; BHB, beta-hydroxybutyrate; HFFD: high fat-fructose diet; LPS: lipopolysaccharide; MWM: Morris water maze; NFD: normal-fat diet; NOR: novel object recognition

As shown in Fig. 1, the study included normal control animals allowed to eat NFD, another group in which rats received HFFD/LPS without treatment, and a third group in which the model animals were administered BHB orally at a dose of 125 mg/kg. The dose was adopted from Torres-Esquivel et al. (2020); however, since i.p. injection caused animal mortality, BHB was administered orally instead. The BHB treatment continued for 7 days starting 3 h after the LPS injection. Animals were distributed equally among the groups, nine rats each.

### Behavioral tests

#### Y-maze test

This experiment evaluates short-term spatial and working memory. The animal’s natural curiosity was demonstrated by their ability to investigate all three arms. Rats with intact working memory recall previously visited arms and prioritize exploring less frequently visited ones. A high percentage of entries into successive arms is interpreted as a significant percentage alternation. A larger percentage of repeated inputs into the same arm indicates a low percentage alternation. To conduct the test, one day after the last dose of BHB (day 8), each rat was placed individually in the middle of the maze and allowed to navigate freely for 120 s, and the alternations number between each arm and arm entry frequency were recorded. Finally, spontaneous alternation percentage (SPA) was computed based on the following formula (Kraeuter et al. 2019):

$${\text{Spontaneous }}\,{\text{alternation}} \% \left( {{\text{SPA}}} \right) = \frac{{{\text{Number }}\,{\text{of }}\,{\text{alternation}}}}{{\left( {{\text{total }}\,{\text{number }}\,{\text{of }}\,{\text{arm }}\,{\text{entries}} {-} 2} \right)}} \times 100$$

#### Morris water maze (MWM) test

Both working and long-term spatial memories were evaluated using the MWM test. The test apparatus consisted of a round basin of 150 cm diameter and a depth of 50 cm. The pool was evenly quartered into four sections labelled as N, S, E, and W, indicating, respectively, north, south, east, and west and each designated with a visual cue. Before the start of the test, the basin was stocked with warm water and a podium was placed in quadrant N above the water surface (2.5 cm). The test consisted of 4 training days after LPS (days 4, 5, 6, and 7) and one probe trial day (day 8). Each animal was lowered gently into water facing the wall of the S quadrant and was left for a maximum of 2 min to reach the podium and stayed on it for 10 s. Animals which cannot reach the platform were directed and allowed to stay for 30 s. The same procedure was repeated for two more trials, starting in a different direction in each trial. The time to reach the podium for each rat in each trial was recorded daily. After 24 h of the last dose of treatment (day 8), the animals performed the probe trial in the absence of the podium, starting from the S quadrant, which is opposite to the podium location. Similarly, the animals were left to explore for 120 s, and the time spent in the target quadrant was recorded (Vorhees and Williams 2006).

#### Novel object recognition (NOR) test

The task assesses the aptitude of the rat to identify the presence of a different object in the field, and since there is no reward or punishment, this method evaluates the original tendency of the rat to explore the new item. This test was carried out on 3 days. On the first day (day 6 of treatment), the animals were left to adapt to the test arena, which consisted of an open field box, for 2 min (habituation phase). On the next day (day 7 of treatment), a single rat was left in the test arena while interacting with two uniform objects (A + A). During this phase (familiarization), the rat was permitted to explore the two undistinguishable objects for at least three sessions (10 min each), and the total exploration time of the final (third) session should be nearly evenly split between the two objects, indicating that the animals had reached a baseline level of interest. In other words, the animals are no longer driven by novelty, and the environment has become familiar to them. On the test phase, which was performed on day 8, each animal was situated in the test environment with a pair of different objects, a familiar one and a novel one (A+B). The animal in this phase was left to investigate the objects for 120 s and the time allocated to examining each object is tracked (Antunes and Biala 2012). Finally, the discrimination (DI) and preference (PI) indices are assessed according to the equations provided below.

$$\begin{aligned} {\text{DI}} & = \frac{{\left[ {{\text{time exploring new object}} - {\text{time exploring familiar object }}} \right]}}{120} \\ {\text{PI}} & = \frac{{\left[ {\text{time exploring new object}} \right]}}{{{\text{time exploring new object}} + {\text{time exploring familiar object }}}} \times 100 \\ \end{aligned}$$

### Collection of blood samples

After 8 weeks on HFFD and once again after LPS injection, rats were fasted for 10 h and anesthetized using thiopental to withdraw a blood sample from the jugular vein. Sera was prepared from the collected blood after being centrifuged for 10 min at 4000 rpm (4 °C). Using the corresponding ELISA kit, serum insulin (Cat# ELR-Insulin; RayBiotech, GA, USA) was assessed, whereas TGs (Cat# MA-TG-1; RayBiotech), TC (Abcam, MA, USA, Cat# ab65390), and glucose (SPINREACT, Girona, Spain, Cat# MD41011) were measured calorimetrically following the guidelines stated in the pamphlet. Finally, HOMA-IR was calculated as stated before.

### Whole brains and hippocampi isolation

On day 8, following the behavioral tests, euthanasia was conducted using thiopental at a dose of 100 mg/kg and in a buffered neutral formalin (10%), three brains/group were fixed for microscopical examination. Meanwhile, the hippocampi were separated from the remaining rats (*n* = 6) for the consequent biochemical analysis.

#### Assessment of LAMP2A, Hsp70, caspase-11, GSDMD-N, IL-1β, IL-18, Ach, and 5-HT, in addition to microglia markers

ELISA technique and MyBioSource diagnostic kits (CA, USA) were used for the determination of hippocampal Hsp70 (Cat# MBS035085), caspase-11 (Cat# MBS008490), N-terminal cleaved gasdermin-D (GSDMD-N; Cat# MBS7255418), NOS2 (Cat# MBS2702569), IL-4 (Cat# MBS355442), Ach (Cat# MBS2700397), and 5-HT (Cat# MBS2700308). Meanwhile, IL-18 (Cat# ab213909; Abcam), nitrated Aβ (Cat# MABN779; Merck, MA, USA), IL-1β (Cat# ELR-IL-1b; RayBiotech), IDE (Cat# E-EL-R2455; Elabscience, TX, USA), and LAMP2A (Cat# 51-2200; ThermoFisher MA, USA) were determined by ELISA technique following the respective kit directions.

#### Western blot detection of cleaved-caspase1 and NLRP3

Total proteins were extracted from the hippocampi homogenates and separated on a gel according to their molecular weight using SDS-PAGE electrophoresis. The separated proteins were collected in a transfer sandwich using PVDF membrane immersed in a transfer buffer. The system was run for 7 min at 25 V to allow protein bands transfer from gel to membrane using Trans-Blot Turbo (BioRad, CA, USA) followed by blocking the membrane in TBST and bovine serum albumin (3%) at room temperature for 60 min. Cleaved caspase-1 (Cat# APB592Ra01; Cloud-Clone Corp, TX, USA), polyclonal NLRP3 (Cat# PA5-79740; ThermoFisher), and beta actin (Cat# ab8227, Abcam) primary antibodies were diluted (1:500) in TBST, added, and held at the appropriate temperature (4°C) for several hours. The membrane was washed by TBST and incubated for another 60 min at room temperature with the secondary antibody (goat anti-rabbit; Novus Biologicals, CO, USA) that was conjugated to HRP. The chemiluminescent substrate (Cat#170-5060; Bio-Rad) was applied following a 2^nd^ rinse in accord to the manufacturer’s instructions and the CCD camera-based imager was used to capture the signals. Finally, ChemiDoc MP imager (Bio-Rad) was used to normalize the band intensities to beta actin.

#### Histopathological and immunohistochemical examinations

Brains fixed in neutral buffered formalin were paraffin wax fixed after being routinely processed to be sliced (5 µm) and stained with either hematoxylin and eosin (H&E) for histopathological examination or Congo red special stain for the detection of amyloid beta (Aβ). Conversely**,** paraffin-embedded tissue blocks were cut on adhesive slides to obtain 5 µm sections for immune staining. Concisely, after being rehydrated and subjected to heat-induced epitope retrieval, tissue sections were blocked for endogenous peroxidases. The primary antibody of anti-Iba (Cat# 10904-1-AP; Proteintech, Planegg-Martinsried, Germany) and phosphorylated Tau (*p*-Tau) (Cat#AP1423; Abclonal, MA, USA) were applied to the tissue sections and left to incubate for 60 min and then washed, and HRP-labeled detection kit (BioSB, USA) was used to develop the reaction. Software of CellSens Dimensions (Olympus) was adopted to examine the stained sections to be quantified as the area percentage relative to negative control slides, prepared by omitting the 1ry antibody, using image J 1.53t, Wayne Rasband and contributors, National Institutes of Health, USA.

### Statistical analysis

All experimental groups were equal by design (*n* = 9), and data were presented in grouped column scatter plots and expressed as mean ± SD. GraphPad prism software, version 8 (GraphPad Software, CA, USA), was used for statistical analysis. Difference between two groups was determined using unpaired Student’s *t* test, whereas comparison between more than two groups was carried out using either ordinary analysis of variance (ANOVA) and Tukey’s multiple comparison test as the *post-hoc* test or Welch’s ANOVA and Dunnett’s T3 multiple comparison test as the *post-hoc* test according to the homogeneity or heterogeneity of variances between groups, respectively, *P* <0.05.

## Results

### Effect of HFFD consumption alone for 8 weeks and in addition to LPS on indicators of IR

As shown in Table 1, 8 weeks on HFFD elicited a significant increase in serum glucose by 27.5%, insulin by 94%, HMOA-IR by 148.8%, TGs by 114%, and TC by 67.6% as compared to NFD group. Meanwhile, the administration of a single LPS dose maintained these disturbances as documented by the increased levels of serum glucose (1.2 folds) and insulin (2.34 folds) that were reflected on the HOMA-IR (2.82 folds) compared to the NFD/saline. This also involves the perturbed lipid profile as verified by the 155.5% and 90.7% elevation in serum TGs and TC, respectively, relative to the NFD/saline group.

**Table 1:** Effect of 8 weeks of HFFD and single LPS injection on fasting serum glucose, insulin, and HOMA-IR, as well as serum TGs and TC

Groups	Week 8		Start of week 9	
	NFD	HFFD	NFD/saline	HFFD/LPS
*Parameters*				
Glucose (mg/dl)	89.17 ± 5.38	113.7 ± 6.89^t^	98.8 ± 9.17	117.5 ± 11.6^t^
Insulin (mIU/ml)	36.35 ± 5.22	70.51 ± 13.78^t^	33.8 ± 7.80	79.13 ± 4.23^t^
HOMA-IR	8.015 ± 1.33	19.94 ± 4.77^t^	8.17 ±1.64	23.0 ±2.61^t^
TGs (mg/dl)	79.10 ± 12.88	169.3 ± 19.69^t^	71.3± 9.67	182.2 ± 4.79^t^
TC (mg/dl)	127.2 ±11.37	213.2 ± 32.49^t^	126.5± 10.07	241.2 ± 13.7^t^

Rats were fed either NFD or HFFD for 8 weeks. NFD group was then injected with saline, and HFFD group was injected with a single dose of LPS (2 mg/kg, i.p) dissolved in saline. Values are expressed as mean (six rats/group) ± SD. Statistical analysis was performed using unpaired Student’s *t* test, *P*<0.05 as compared with (t) NFD group or NFD/saline. *HFFD* High-fat fructose diet, *HOMA-IR* Homeostasis model assessment of IR, *NFD* Normal-fat diet, *TC* Total cholesterol, *TGs* Triglycerides

### BHB enhances short- and long-term spatial working memory in addition to recognition memory in HFFD/LPS-induced AD-like rats

**Fig. 2:**
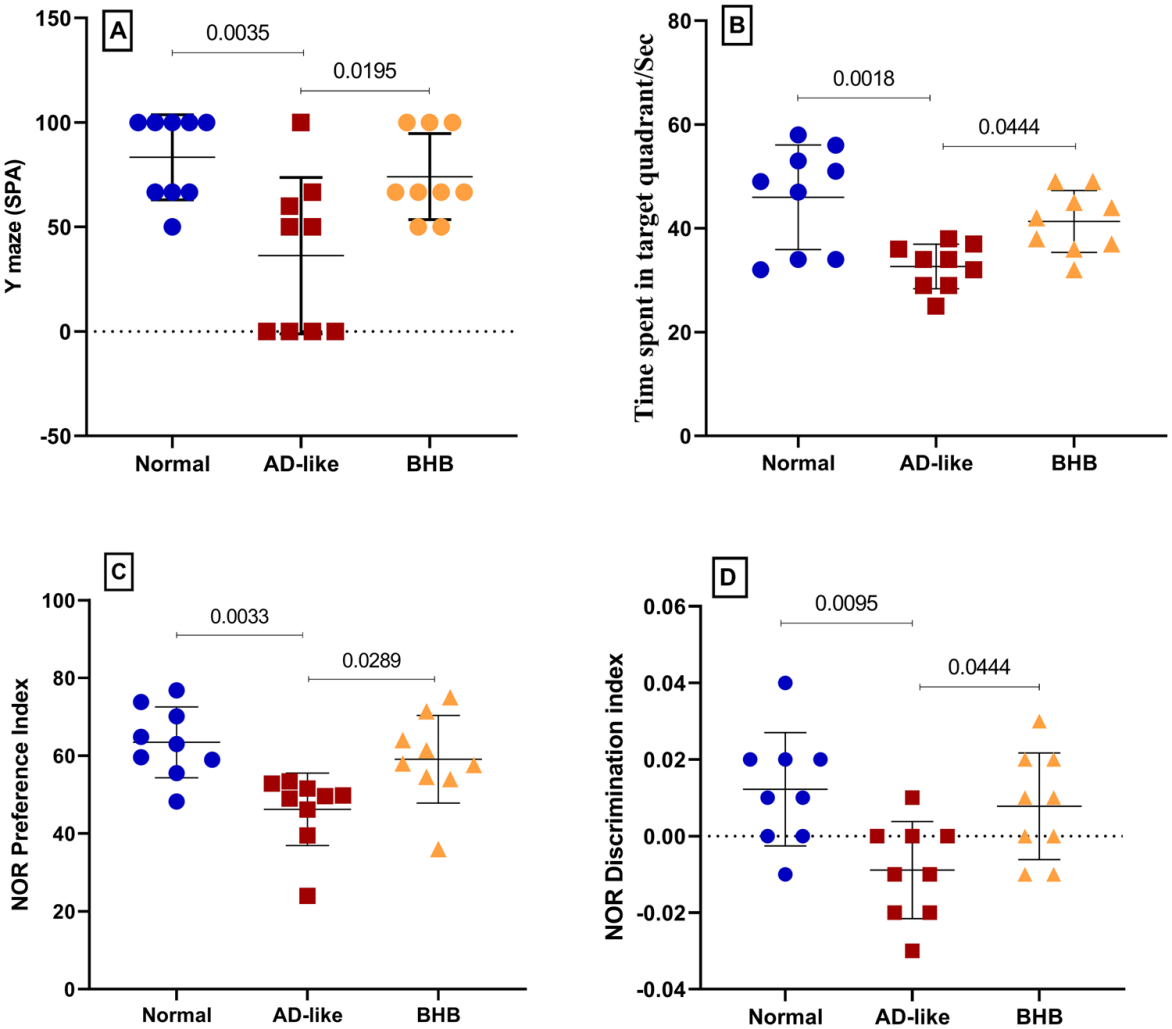
BHB post-administration enhanced the performance of HFFD/LPS-induced AD-like rats using (**A**) Y-maze, (**B**) MWM, and (**C** and **D**) NOR tests. The results are presented as scatter plot and depicted as mean ± SD of nine rats/group. One-way ANOVA was adopted for statistical analysis, and the *post-hoc* test was the Tukey’s multiple comparison test; *P*<0.05. AD: Alzheimer’s disease; BHB, beta-hydroxybutyrate; DI: discrimination index; MWM: Morris water maze; NOR: novel object recognition; PI: preference index

As shown in Fig. 2, AD-like rats displayed reduced proficiency in the (A) %SPA and the (B) seconds spent in the intended quadrant, reflecting the deterioration in both short- and long-term working memories, respectively. Furthermore, a decrease in animals’ (C) PI and (D) DI was also observed in the NOR test which reveals a decline in recognition memory. Treatment with BHB for 7 days was able to normalize the animal’s performance in all tests.

### BHB ameliorated hippocampal Aβ and p-Tau deposition in HFFD/LPS-induced AD-like rats

**Fig. 3:**
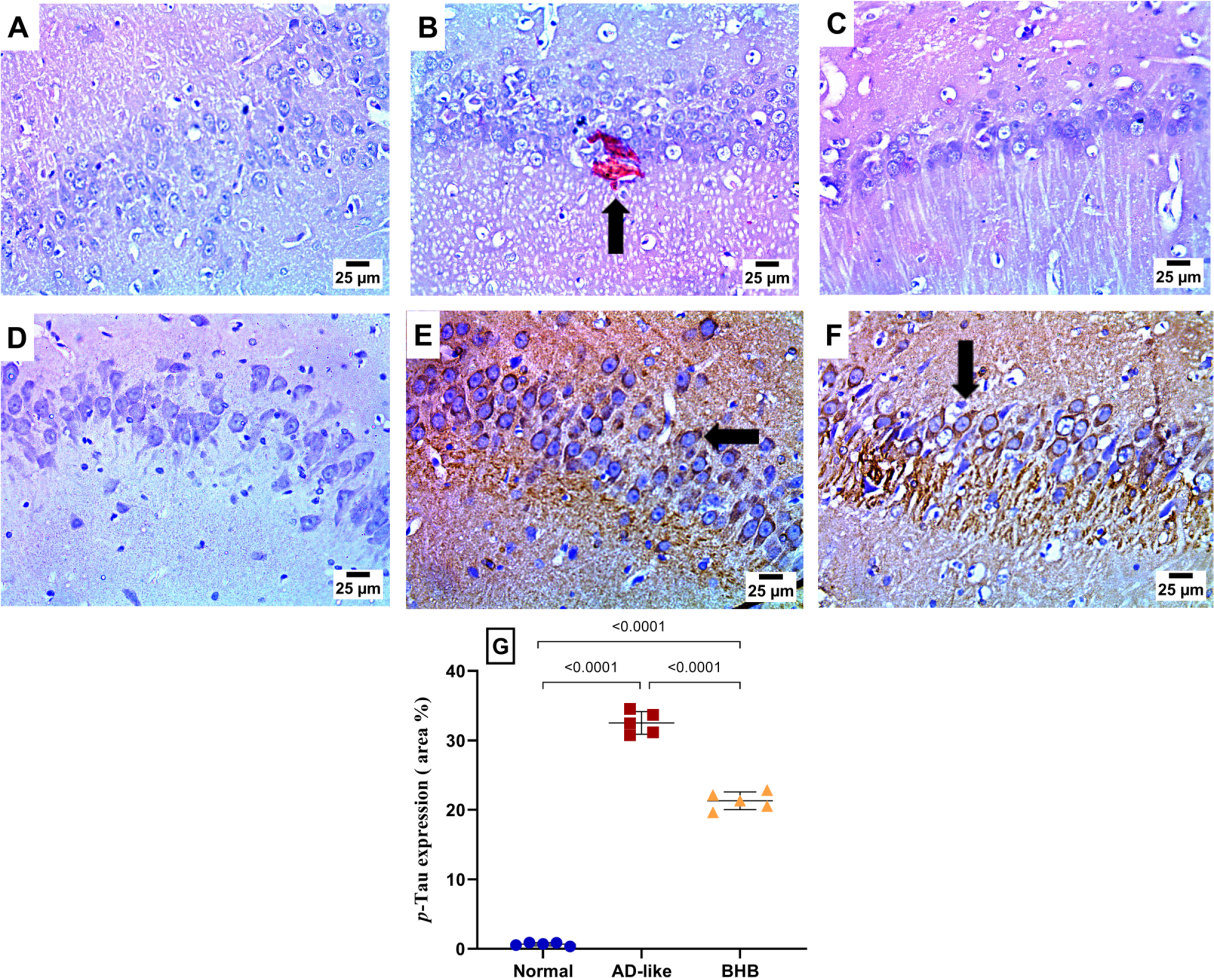
BHB post-administration ameliorated hippocampal (**A**) Aβ and (**B**) *p*-Tau deposition in HFFD/LPS-induced AD-like rats. As visualized by the Congo red stain, Aβ aggregates were detected in the hippocampus of (**B**) AD-like group, as compared to the (**A**) normal group. However, Aβ aggregates were not detected in the (**C**) BHB-treated group (scale bar 25 μm). Meanwhile, the immune-stained section of (**E**) AD-like group reveals increased *p*-Tau-positive staining compared to the (**D**) normal group. Similarly (F) BHB-treated group displayed a profound decrease in the immunoexpression of *p*-Tau. The % area of positive *p*-Tau staining is presented in panel (**G**). The results are presented as scatter plot and depicted as mean ± SD of five random fields of three rats/group. One-way ANOVA was adopted for statistical analysis, and the *post-hoc* test was the Tukey’s multiple comparison test; *P*<0.05. AD: Alzheimer’s disease; Aβ: amyloid beta; BHB, beta-hydroxybutyrate; *p*-Tau: phosphorylated tau

Figure 3 delineates the expression of the AD hallmarks. As presented in panel B, Congo red staining confirmed the expression of Aβ in the AD-like cohort, compared to the normal control group. In contrast, the BHB-treated group exhibited a negative reaction to the stain, indicating the absence of this hallmark. In a similar pattern, section E demonstrated extensive immunoexpression of hyperphosphorylated Tau, a pathological protein linked to neurodegeneration, whereas the normal group showed no expression. Notably, BHB administration attenuated Tau expression, highlighting its neuroprotective potential.

### BHB increased hippocampal contents of LAMP2A and Hsp70 in HFFD/LPS-induced AD-like rats

**Fig. 4:**
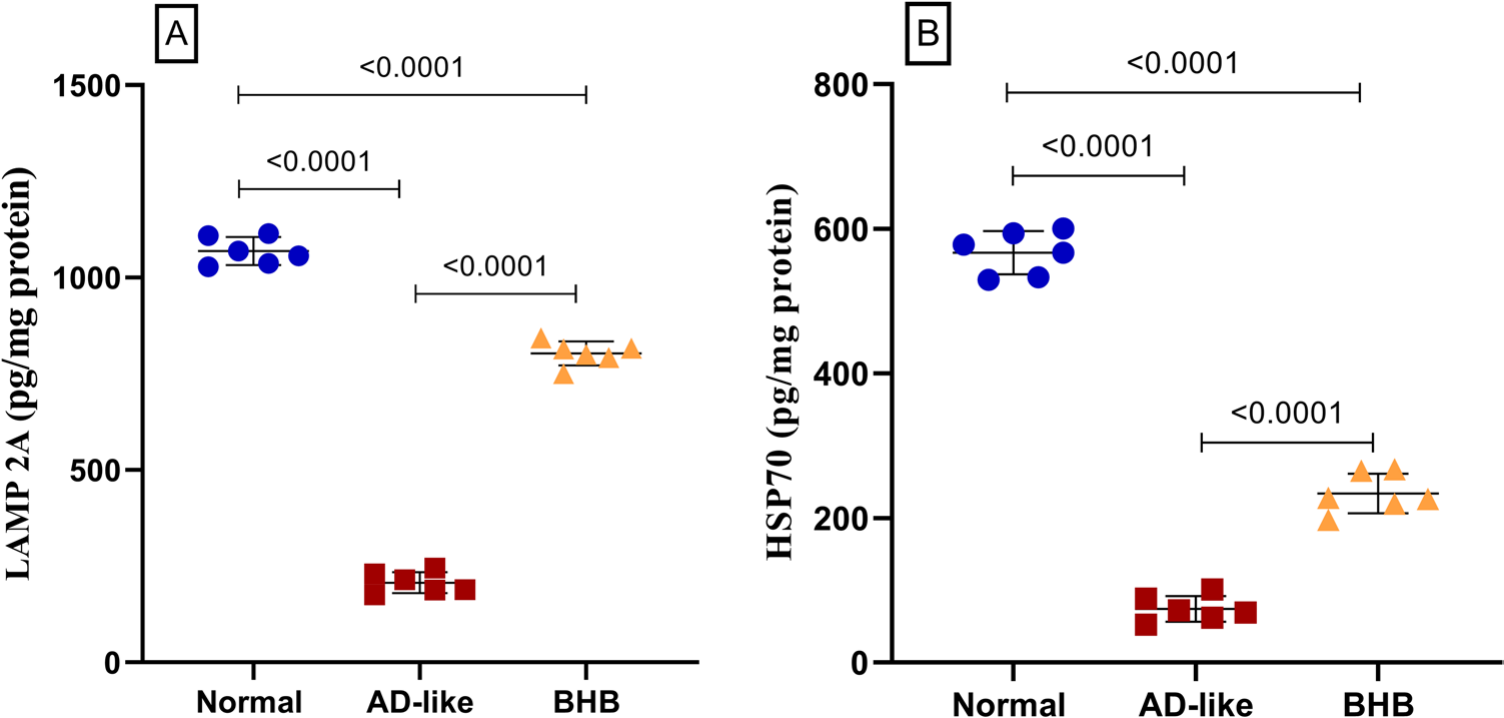
BHB post-administration enhanced the hippocampal contents of (**A**) LAMP2A and (**B**) Hsp70 in HFFD/LPS-induced AD-like rats. The results are presented as scatter plot and depicted as mean ± SD of six rats/group. One-way ANOVA was adopted for statistical analysis, and the *post-hoc* test was the Tukey’s multiple comparison test; *P*<0.05. AD: Alzheimer’s disease; BHB, beta-hydroxybutyrate; Hsp70, heat shock protein 70; LAMP2A, lysosomal-associated membrane protein 2A

As one of the CMA markers, Fig. 4 illustrates that the HFFD/LPS model caused a sharp dwindling in the hippocampal content of (A) LAMP2A, an integral rate-limiting protein in the CMA process, reducing it to only 19% of normal control values. Likewise, (B) Hsp70, a chaperone protein, was depleted in the AD-like group to 13% of normal levels. In contrast, post-administration of BHB replenished LAMP2A content by fourfold and Hsp70 by 3.2-fold compared to the AD-like group.

### BHB alleviated hippocampal canonical inflammasome markers in HFFD/LPS-induced AD-like rats

**Fig. 5:**
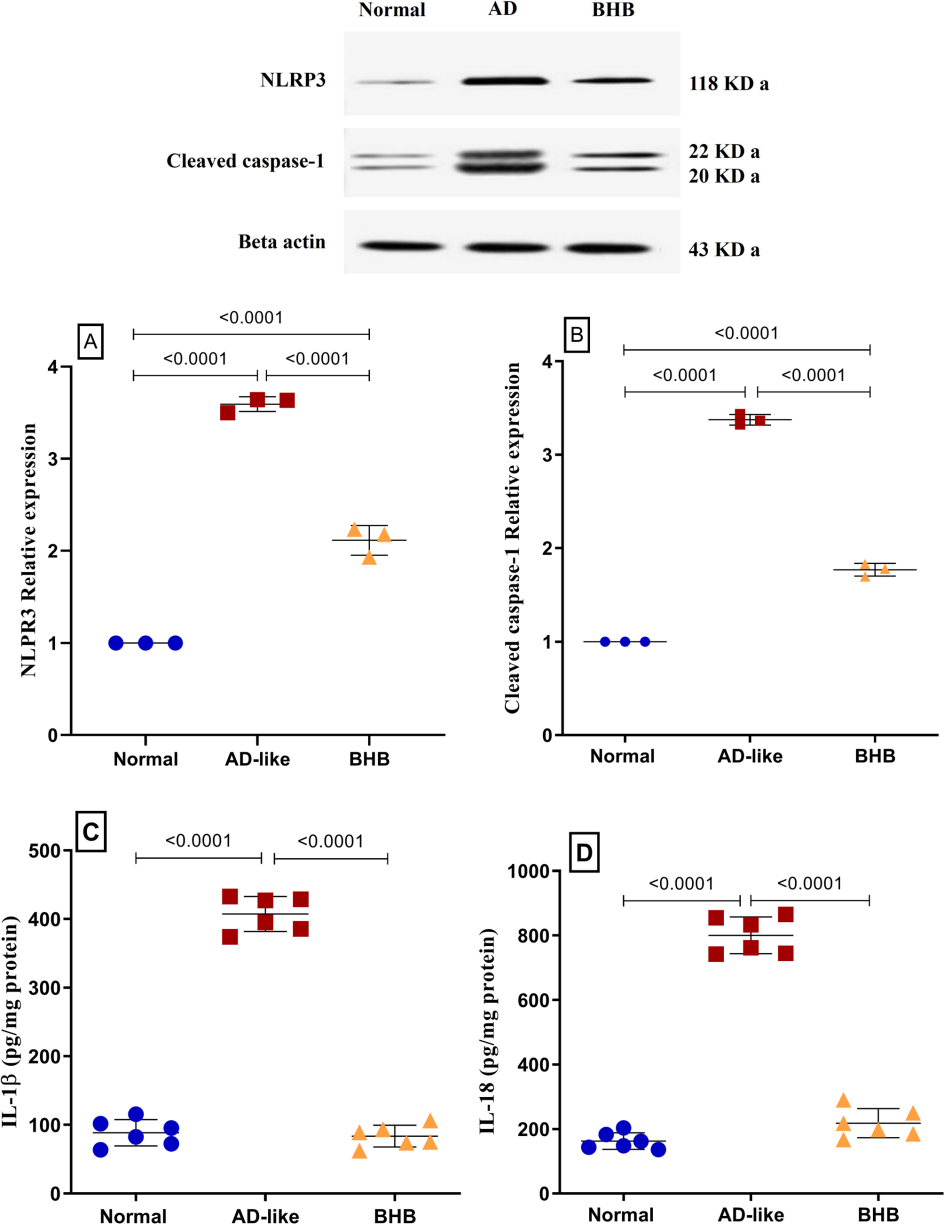
BHB post-administration alleviated the hippocampal protein expression of inflammasome markers in HFFD/LPS-induced AD-like rats: (**A**) NLRP3 and (**B**) cleaved caspase-1 and content of (**C**) IL-1β and (**D**) IL-18 in rats. The results are presented as scatter plot and depicted as mean ± SD of three (WB) or six (ELISA) rats/group. One-way ANOVA was adopted for statistical analysis, and the *post-hoc* test was the Tukey’s multiple comparison test; *P*<0.05. AD: Alzheimer’s disease; BHB, beta-hydroxybutyrate; NLRP3, nucleotide-binding domain, leucine-rich–containing family, pyrin domain–containing-3

Figure 5 displays the association between HFFD/LPS-induced AD-like model and inflammatory canonical inflammasome. Relative to control group, this verity was perceived by the marked upregulated protein expression of (A) NLRP3 and (B) cleaved caspase-1 by more than 3-fold. In turn, both inflammatory cytokines (C) IL-1β and (D) IL-18 were boosted by 360% and 392%, respectively. However, relative to the insult, BHB treatment reduced NLRP3 and cleaved caspase-1 by 41.1% and 47.6%, respectively, to restore the inflammatory cytokines back to their normal level.

### BHB abated hippocampal non-canonical inflammasome-induced pyroptosis in HFFD/LPS-induced AD-like rats

**Fig. 6:**
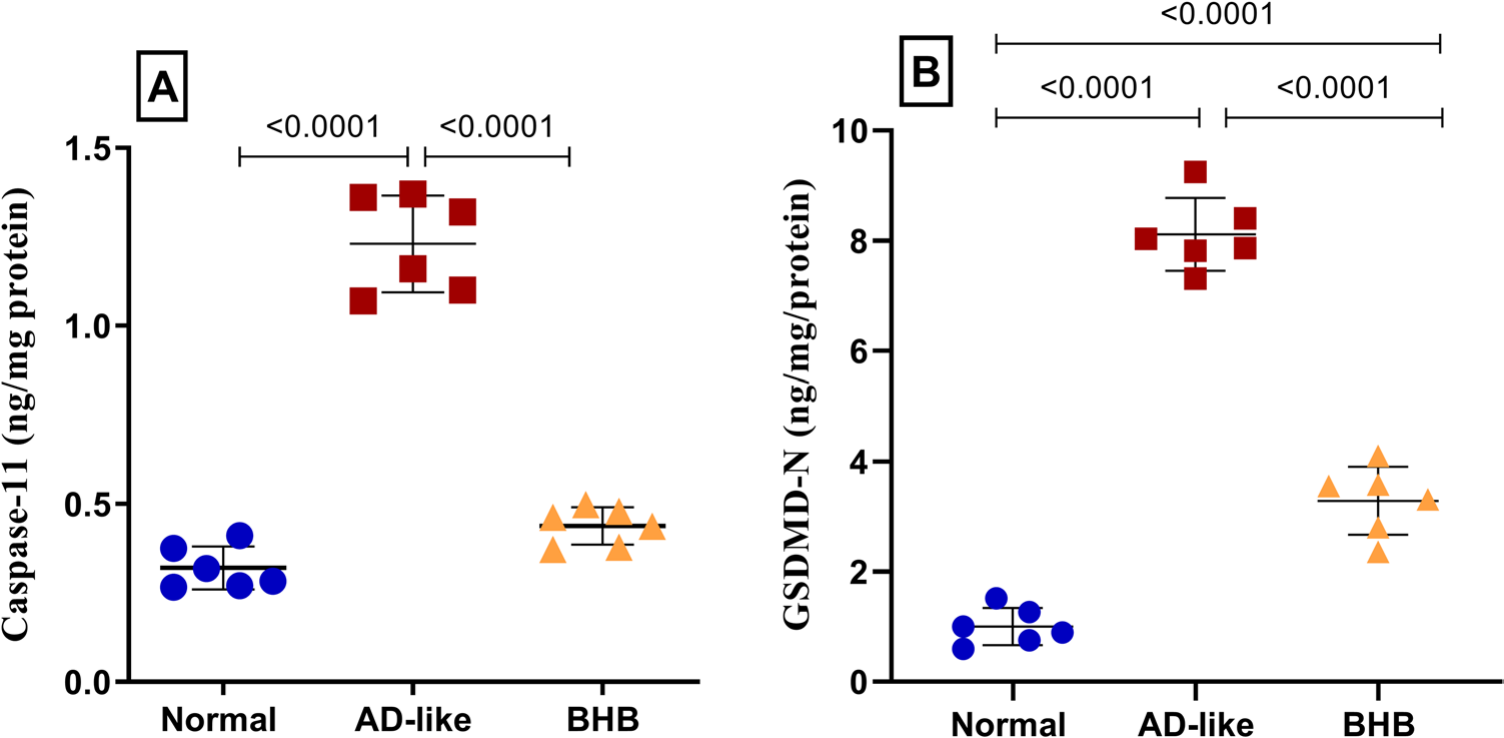
BHB post-administration abated the hippocampal content of apoptotic markers in HFFD/LPS-induced AD-like rats: (**A**) caspase-11 and (B) GSDMD-N. The results are presented as scatter plot and depicted as mean ± SD of six rats/group. One-way ANOVA was adopted for statistical analysis, and the *post-hoc* test was the Tukey’s multiple comparison test; *P*<0.05. AD: Alzheimer’s disease; BHB, beta-hydroxybutyrate; IL-1β, interleukin-1 beta; IL-18, interleukin-18

The current model caused neuronal cell pyroptosis as presented in Fig. 6. In this regard and partly in the presence of LPS, the current model bolstered hippocampal pyroptotic markers, (A) caspase-11 and (B) GSDMD-N by 3.8- and 8-fold, respectively, as compared to the normal value. Nevertheless, these markers were notably inhibited in the BHB-treated group, as caspase-11 was restored to its normal value and GSDMD-N was abated by 60% relative to the HFFD/LPS model.

### BHB skewed hippocampal microglia toward the protective M2 phenotype in HFFD/LPS-induced AD-like rats

**Fig. 7:**
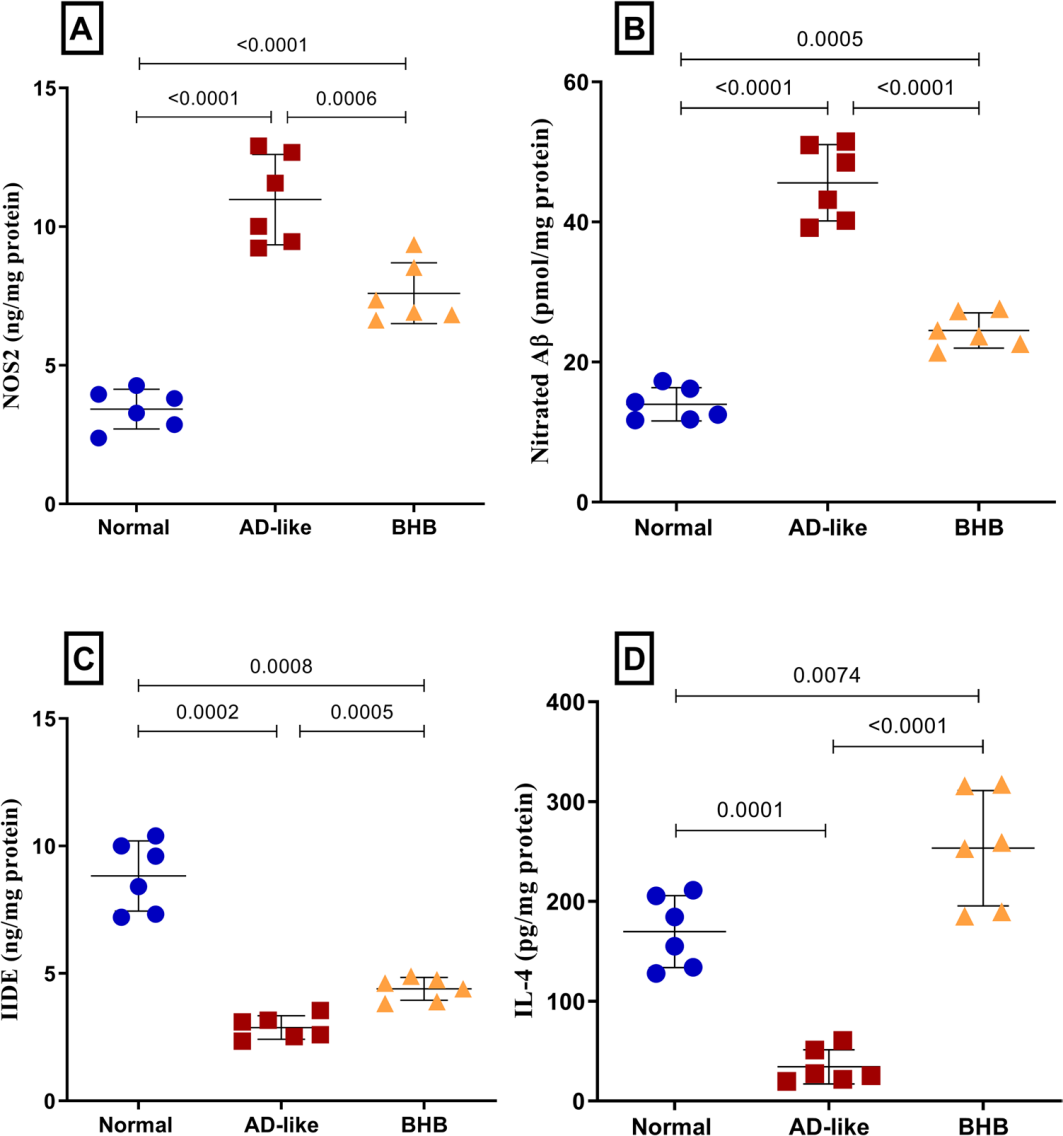
BHB treatment for 7 days diverted hippocampal microglia to M2 phenotype in HFFD/LPS-induced AD-like rats: (**A**) IDE, (**B**) IL4, (**C**) NOS2, and (**D**) nitrated Aβ in rats. The results are presented as scatter plot and depicted as mean ± SD of six rats/group. One-way ANOVA was adopted for statistical analysis, and the *post-hoc* test was the Tukey’s multiple comparison test, while Welch’s ANOVA followed by Dunnett’s T3 multiple comparison as the *post-hoc* test was used for IDE. *P*<0.05 was accepted for both tests as the least significant level. AD: Alzheimer’s disease; BHB, beta-hydroxybutyrate; IDE, insulin-degrading enzyme; IL-4, interleukin-4; NOS2, nitric oxide synthase 2

**Fig. 8:**
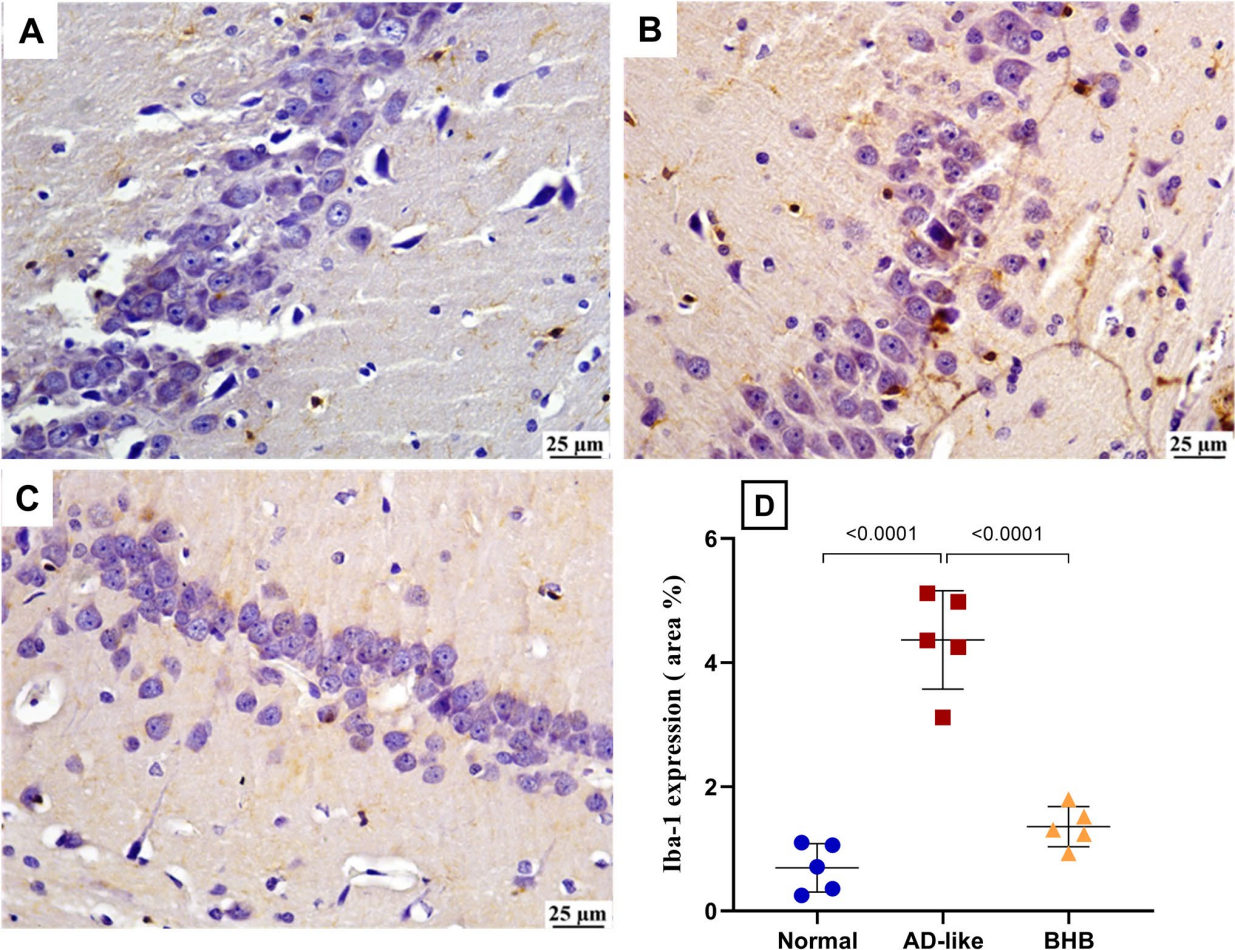
BHB treatment for 7 days reduced hippocampal Iba-1 immunoexpression in HFFD/LPS-induced AD-like rats. As depicted in the hippocampus region of the (**B**) AD-like group, Iba-1 was significantly immunoexpressed relative to that of the (**A**) normal group, which depicts limited Iba-1 expression. However, treatment with (**C**) BHB-treated group reveals mild Iba-1 staining (scale bar 25 μm). The % area of positive Iba-1 staining is presented in panel (**D**). The results are presented as scatter plot and depicted as mean ± SD of five random fields of three rats/group. One-way ANOVA was adopted for statistical analysis, and the *post-hoc* test was the Tukey’s multiple comparison test; *P*<0.05. AD: Alzheimer’s disease; BHB, beta-hydroxybutyrate; Iba-1, Ionized calcium-binding adaptor molecule-1

Figure 7 shows that AD-like model induction triggered the hippocampal microglia polarization toward the M1 phenotype while diminishing the protective M2 microglia. Being markers of M1-activated microglia, the current insult sharply elevated (A) NOS2 and (B) nitrated Aβ to 3.2-fold and 3.3-fold, respectively, relative to the normal value. In addition, the model heavily reduced the M2 microglia-related parameters evident by (C) 67.3% reduction in IDE and an (D) 79.8% decline in IL-4. Conversely, BHB treatment reduced NOS2 by 30.8% with consequent reduction in the nitrated Aβ by 46.2%, while modestly enhancing IDE (1.5-fold) and extremely elevated IL-4 (7.4-fold) to exceed the normal control value. In the same milieu, microglia activation was further documented in Fig. 8. As depicted in the (B) AD-like group, a significant expression in Iba-1 is noted relative to the (A) normal group. Nevertheless, post-treatment with (C) BHB lessened this expression as compared to AD group.

### BHB restored hippocampal content of the neurotransmitters Ach and 5-HT in HFFD/LPS-induced AD-like rats

**Fig. 9:**
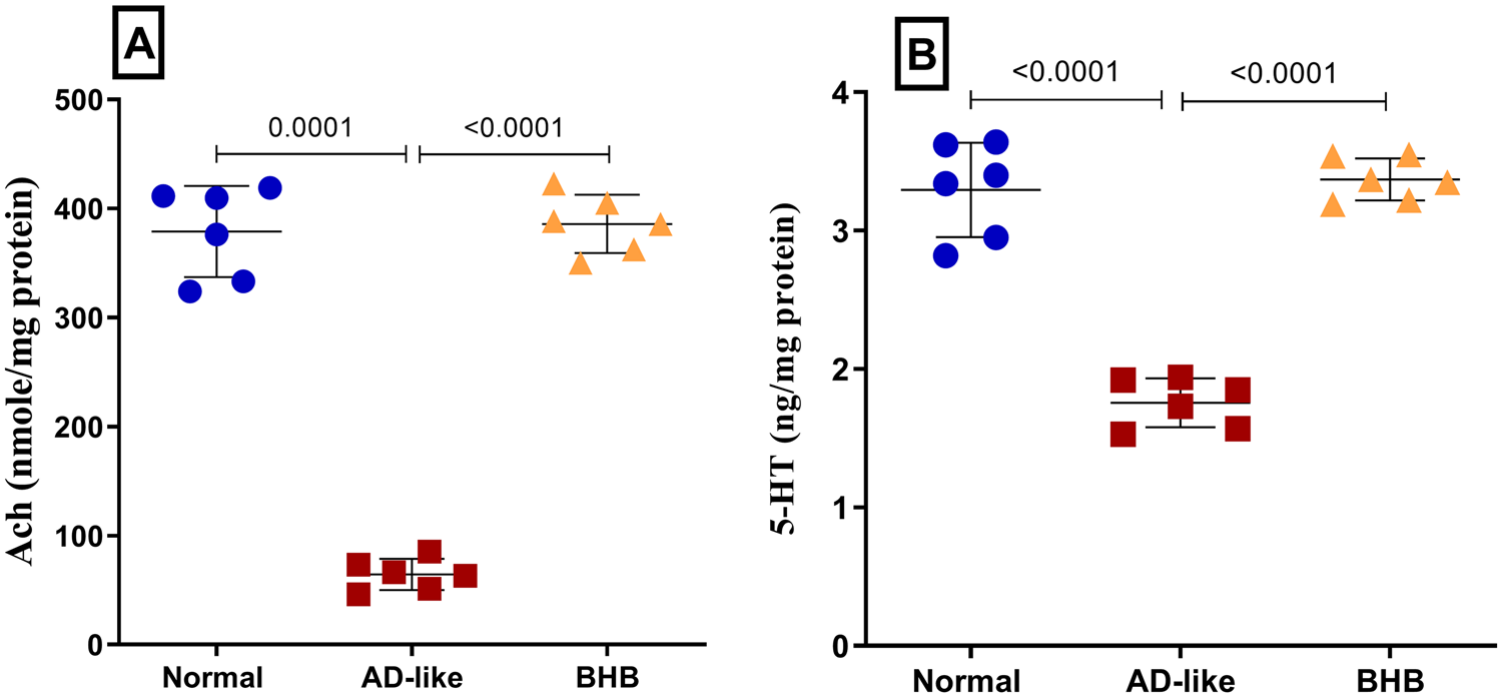
BHB post-administration restored the hippocampal content of (**A**) Ach and (**B**) 5-HT in HFFD/LPS-induced AD-like rats. The results are presented as scatter plot and depicted as mean ± SD of six6 rats/group. One-way ANOVA was adopted for statistical analysis, and the *post-hoc* test was the Tukey’s multiple comparison test; *P*<0.05. 5-HT: serotonin; Ach: acetylcholine; AD: Alzheimer’s disease; BHB, beta-hydroxybutyrate

Figure 9 highlights the profound neurotransmitter dysregulation associated with the disease model, where a substantial depletion of both (A) Ach and (B) 5-HT was detected in the AD-like model, with reductions of 83% and 47%, respectively, as compared to the normal group. However, the potential neuromodulatory role of BHB in counteracting AD-like neurotransmitter and cognitive deficits is supported by the effective restoration of both neurotransmitters following the post-administration of BHB for 1 week, which brought them back to near-normal values.

### BHB improved hippocampal histopathological picture altered by the HFFD/LPS model

**Fig. 10:**
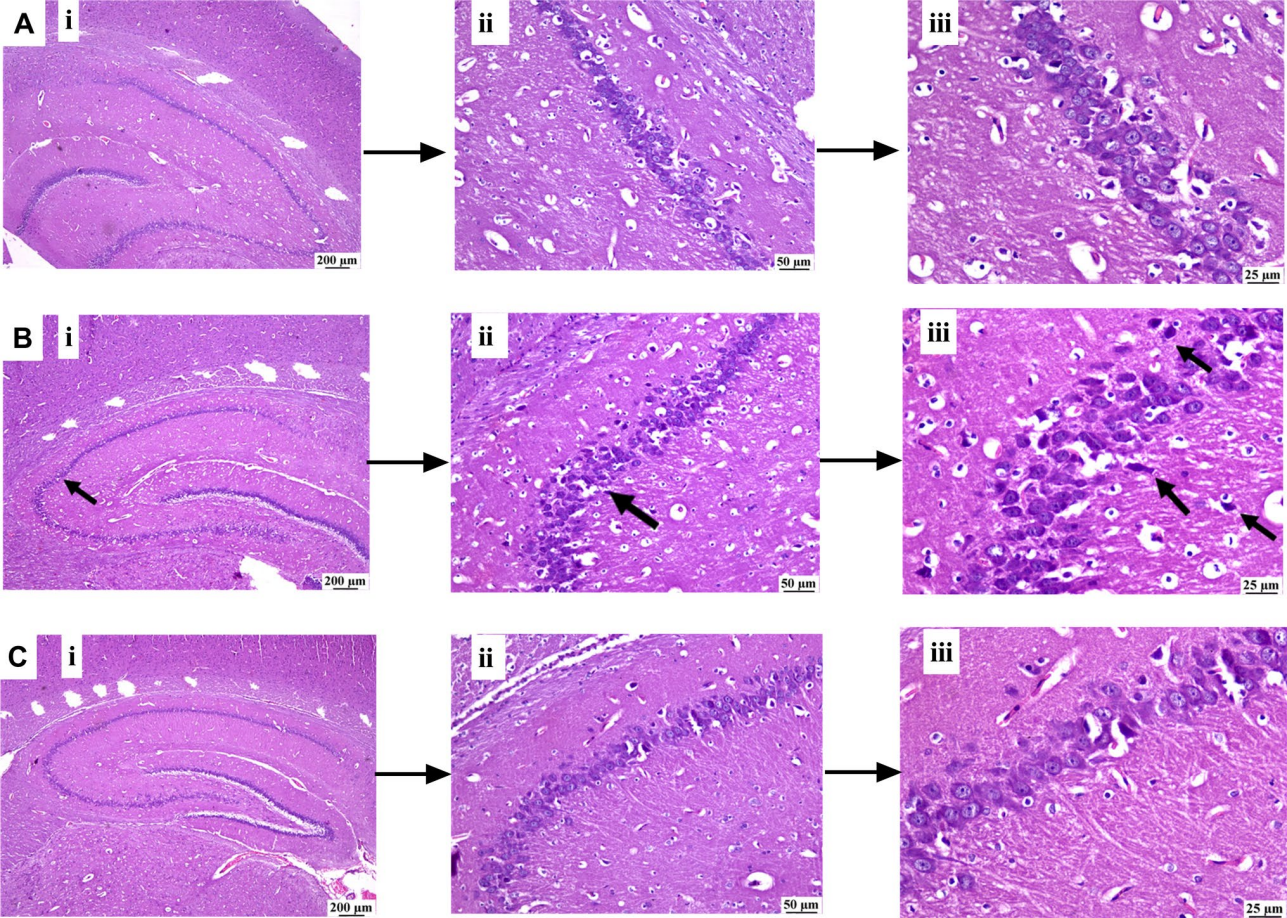
Effect of 8-day administration of BHB on hippocampal histopathological picture in rats. As compared to H&E-stained hippocampal section of (**A**) the normal group, which shows normal hippocampus, the section of (**B**) AD-like group reveals some degenerating neurons within the hippocampus (arrow) with gliosis. On the other hand, the section of (**C**) BHB-treated group shows restored normal hippocampal architecture (scale bar: [i] 200 μm, [ii] 50 μm, and [iii] 25 μm)

Microscopic examination of brain sections (Fig. 10) from (A) the normal control group reveals the original structure of the hippocampal region. However, (B) the AD-like group exhibits dark degenerating neurons within the hippocampus along with gliosis. In contrast, (C) the BHB-treated group shows marked improvement, with the hippocampus appearing normal in the examined sections.

## Discussion

Our findings underscore the ability of BHB to ameliorate HFFD/LPS-induced behavioral, histological, and biochemical perturbations in the rat hippocampus. BHB treatment restored the animals’ working (Y-maze and MWM) and recognition (NOR) memory, reduced Aβ and *p*-Tau deposition, and increased CMA activity (LAMP2A) and the hippocampal content of the neuroprotective chaperone Hsp70. In addition, it halted canonical NLRP3 inflammasome assembly and the non-canonical inflammasome axis (caspase-11/ GSDMD-N) to hinder pyroptosis. Moreover, BHB administration skewed microglia from the inflammatory M1 phenotype verified by the reduced markers (NOS2, nitrated Aβ, and Iba-1 expression) toward the protective M2 phenotype (IDE, IL-4). BHB also normalized the hippocampal contents of Ach and 5-HT and finally restored the hippocampal architecture.

In our study, treatment with BHB enhanced CMA after being curbed by the used model, a finding that, to the authors’ knowledge, has not been previously determined. CMA is among the first cellular signals studied for clearing protein accumulations (Kaushik and Cuervo 2018) and alleviating neuroinflammation in neurodegenerative diseases such as AD (Saxena et al. 2025). In CMA, cytosolic chaperones target specific soluble proteins for lysosomal degradation, thereby helping maintain cellular homeostasis. Unlike other autophagy pathways, CMA transports its substrates to the lysosomal lumen via the transmembrane receptor LAMP2A (Bejarano and Cuervo 2010), rather than being engulfed. Therefore, the expression of LAMP2A protein is crucial for regulating CMA activity (Bandyopadhyay et al. 2008), as antagonizing this receptor is still the most focused method for inhibiting CMA (Kaushik and Cuervo 2018; Massey et al. 2006). Previously, an in-vitro study using human embryonic fibroblasts demonstrated that ketone bodies, including BHB, can abet CMA to perform more efficiently by oxidizing substrates to become better recognized by the CMA machinery and translocate to the lysosomal membrane for degradation (Finn and Dice 2005). More recently, Jafari et al. (2024) recounted that aged mice fed with a calorie-restricted diet exhibited sustained CMA upregulation, as evidenced by increased lysosomal LAMP2A levels, the key regulatory component of CMA, partly by stabilizing this protein at the lysosomal membrane. These findings brace our results in which post-administration of BHB activated CMA and elevated LAMP2A levels to counteract the effects of HFFD/LPS. Indeed, HFFD/LPS intake led to CMA suppression, consistent with previous findings, where mice fed with high dietary lipid have been shown to inhibit CMA in the liver (Rodriguez-Navarro et al. 2012), as well as the hypothalamus (Portovedo et al. 2020). The inverse connection between CMA and AD pathological markers has been indicated, where mutant variants of Tau that drive Tau hyperphosphorylation (Iqbal et al. 2010) have been found to bind to LAMP2A to impair CMA activity (Wang and Lu 2022), which in turn fosters further accumulation of *p*-Tau in a self-perpetuating cycle (Wang et al. 2009). Nevertheless, the reactivation of CMA activity attenuates *p*-Tau expression, as demonstrated by BHB treatment in the current study. In this regard, a prior study chronicled that amyloidogenic precursor protein (APP) and *p*-Tau, which are pivotal in the etiopathogenesis of AD, harbor KFERQ motifs, an attribute that facilitates their binding to LAMP2A to be marked for proteolytic degradation via CMA (Wang et al. 2009). Moreover, Xu et al. (2021) stated that activated CMA profoundly quell accumulated Aβ, verities that back our findings.

Hsp70 is a molecular chaperone involved in protein homeostasis that ensures proteins are safeguarded from cellular stress during complex assembly while also preventing their aggregation and disassembly (Mayer and Bukau 2005). In the same milieu, earlier studies underscored the neuroprotective capacity of this protein against thermal (Mailhos et al. 1994) and ischemic (Amin et al. 1996) stresses. In our study, we first showed that BHB has increased hippocampal content of Hsp70 after being nullified by the HFFD/LPS-induced AD-like model, representing one possible hinge for the attenuated Aβ and *p*-Tau expression recorded in this study. In brace to our findings, Moyano et al. (2020) stated that repeated administration of paraquat increased AD hallmarks, caused hippocampal neuronal demise, and impaired cognitive function by downregulating Hsp70. On the other hand, increased Hsp70 has been found to mitigate AD hallmarks by interacting with APP, disrupting its secretory pathway, thereby reducing Aβ formation (Hoshino et al. 2011) and aggregation (Maiti et al. 2014). These co-authors also underscored the ability of increased Hsp70 in promoting the degradation of Tau and Aβ oligomers through the proteasome system (Maiti et al. 2014), highlighting its potential effect in the disease’s pathobiology.

In addition, we found that BHB, recognized as an ATP substitute for the brain (Cunnane et al. 2011), has mitigated neuroinflammation by modulating inflammasome signaling. Specifically, BHB reduced NLRP3 protein expression along with its downstream effector, cleaved caspase-1, leading to a decline in IL-1β and IL-18. Furthermore, BHB inhibited the non-canonical arm of inflammasome by decreasing caspase-11, which is mainly activated by LPS, to ultimately lead to pyroptotic cell death supported by the lowering of GSDMD-N. Our results support previous findings in which BHB was found to impede NLRP3 inflammasome activation in response to multiple chemically distinct NLRP3 activators (Youm et al. 2015), in a 5XFAD mouse model of AD (Shippy et al. 2020), and recently in uric acid-treated murine muscle cell culture (Remund et al. 2024). Although the potential mechanisms underlying BHB-mediated NLRP3 inhibition were recently detailed (Shippy et al., 2025), we propose that CMA activation may serve as an additional pathway contributing to this inhibitory effect. To support this notion, LAMP2A knockout in mice has been associated with increased NLRP3 inflammasome and accelerated atherosclerosis, whereas restoring LAMP2A level greatly attenuated NLRP3 inflammasome activation, denoting NLRP3 as a CMA substrate (Qiao et al. 2021). The same researchers recounted that LAMP2A-mediated change in NLRP3 activity was due to CMA-driven degradation of the NLRP3 protein rather than alterations in its mRNA levels (Qiao et al. 2021). These verities were determined in our study, where a marked decline in hippocampal LAMP2A content in AD rats was associated by enhanced NLRP3/cleaved caspase-1/ IL-1β and IL-18 trajectory. Conversely, BHB treatment effectively reversed these changes, with normalization of inflammatory cytokines. Moreover, the aptitude of BHB to increase Hsp70 represents another mechanism contributing to inflammasome inhibition. Martine et al. (2019) annotated that Hsp70 knockdown led to overactivation of NLRP3, caspase-1, and IL-1β, both murine bone marrow-derived macrophages primed with LPS and peritonitis mouse model. These findings align with our results, in which diminished hippocampal Hsp70 content was evident in the AD group alongside an increased NLRP3 cascade; however, BHB post-administration increased Hsp70 while abating the inflammasome trail. In the same regard, the inhibitory role of Hsp70 in NLRP3 regulation has been reported in acute kidney injury models (Ullah et al. 2020), sepsis-induced cardiomyopathy (Song et al. 2022), and microglia pyroptosis-induced post-operative cognitive dysfunction (Lin et al. 2024).

In fact, the role of microglia cannot be overlooked, as activated microglia participate in neuroinflammation, partly by activating NLRP3, which is canonically stimulated when extracellular LPS and aggregated Aβ1-42 bind to microglial TLR4, initiating persistent activation of microglia (Yang et al. 2020; Yang et al. 2019). In our study, the used rat model increased Aβ in the hippocampus to promote M1 phenotype activation abetted by the increased NLRP3, which was validated by the increased activity of NOS2 and nitrated Aβ, as well as the upregulation of Iba-1 expression in the current hippocampal sections of AD rats, consistent with a previous finding (Heneka et al. 2013). The latter co-authors reported that deficient NLRP3 and caspase-1 resulted in decreased NOS2 and nitrated Aβ. In contrast, BHB-treated sections exhibited reduced Iba-1 staining, along with decreased NOS2 and nitrated Aβ, implying diminished M1 microglia activation, which aligns with the inhibition of the canonical inflammasome trajectory. Furthermore, BHB modulated microglial function by shifting the polarization of microglia toward the protective non-inflammatory M2 phenotype, reversing the AD-induced upregulation of M1 neurotoxic markers. BHB treatment also increased IL-4 and IDE, an enzyme that is responsible in part to Aβ clearance. The ability of BHB to stimulate Hsp70 may contribute to the activation of IDE, as prior study has shown that Hsp70 inhibits Aβ accumulation through enhancing the expression of IDE (Lu et al. 2014), which concurs with our findings. This shift in microglial polarization is significant because the M2 phenotype is associated with neuroprotective functions, including enhanced clearance of amyloid plaques and reduced inflammatory responses (Mosher and Wyss-Coray 2014). By promoting a shift toward the M2 phenotype, BHB helps protect the brain from inflammation and neuronal degeneration, further backing its potential capability as an AD therapy.

Our findings also revealed that treatment with BHB returned both Ach and serotonin to their normal levels after being markedly inhibited in the insult model, results that further pin down the neuroprotective effect of BHB. The mechanism of BHB on Ach could be explained by the aptitude of BHB to act as an acetyl moiety precursor for Ach (Sterling et al. 1981) to impact hippocampal memory, which is lost in AD (H Ferreira-Vieira et al. 2016). Moreover, Hsp70 may be responsible for the increased Ach level as reported in our findings and earlier in rat hippocampus (Frinchi et al. 2018). In addition, it was reported that Ach suppressed LPS-induced neuroinflammation and inflammatory microglia as shown herein and previously (Li et al. 2019) by the activation of its receptor 7α-nAchR.

Indeed, the role of serotonin in regulating neuroinflammation and microglia function is interesting. Alike Ach effect, a previous study reported that the presence of serotonin suppressed LPS-induced pro-inflammatory cytokine secretion (Krabbe et al. 2012), whereas increased hippocampal serotonin level in rats decreases following LPS-induced neuroinflammation (Carabelli et al. 2020). The AD-like mediated neuroinflammation by triggering inflammasome and the occurrence of pyroptosis, driven by inflammasome-associated caspases-1 and -11, events that could contribute to neurodegeneration, ultimately leading to reduced hippocampal levels of both Ach and serotonin. Contrariwise, it can be postulated that BHB, by inhibiting both caspases and subsequently suppressing inflammation and pyroptosis, may partly explain the restoration of both neurotransmitters’ levels. Similar to the Ach-mediated decrease I inflammatory microglia, brain serotonin-microglia interaction revealed that exhaustion of 5-HT-releasing neurons amplified microglial density as detected by increased Iba-1 expression (Vetreno et al. 2017) to concur with our results. Furthermore, the absence of microglial serotoninergic receptor 5-HT2b prolonged LPS-induced neuroinflammation and aggravated cytokine expression (Béchade et al. 2021).

Collectively, these investigations point to the role of Ach and serotonin in retaining microglia in their anti-inflammatory resting state, backing our results, where AD-like insult, as shown herein, displayed lower hippocampal content of both neurotransmitters along with increased immunoexpression of Iba-1, as well as hippocampal contents of NOS2 and nitrated Aβ, indicating the tilting of microglia toward pro-inflammatory M1 polarization. Hence, besides the role of Ach in alleviating AD characteristics, the current findings reported, for the first time, that serotonin also can be nominated as a novel therapeutic target for BHB to alleviate AD pathology.

BHB treatment markedly enhanced cognitive function, as perceived by enhanced performance in memory tests (Y-maze, MWM, and NOR), suggesting its neuroprotective role, likely due to its function as an alternative brain energy source (Cunnane et al. 2016). Hyperinsulinemia and IR and, observed in this study and previously (Ferreira et al. 2018), impair glucose utilization while also limiting ketone production due to insulin’s anti-lipolytic effect, contributing to cognitive decline (Cunnane et al. 2011). This might elucidate the procognitive potential of BHB by serving as an alternative ATP source. In addition, excess insulin competes for IDE, an enzyme crucial for Aβ clearance (Farris et al. 2003), potentially exacerbating Aβ accumulation, as observed in our findings.

## Conclusion

Overall, the presented results highlight the valuable impact of BHB in mitigating AD pathology through the restoration of CMA activity, Hsp70 content, promotion of neuroprotective microglial activity, amelioration of neuroinflammation, and restoration of 5-HT and Ach contents to collectively inhibit Aβ and p-Tau. The findings, hence, nominate BHB as a multifaceted treatment option for improving mental function and slowing the progression of AD, unveiling CMA, Hsp70, and 5-HT as possible therapeutic targets for the treatment of sporadic AD.

## Supplementary Information

Below is the link to the electronic supplementary material.

Supplementary file1 (PDF 179 kb)

## Data Availability

The datasets generated during and/or analyzed during the current study are available from the corresponding author on reasonable request.
