# Supplementary material for: Chaperone-mediated autophagy, heat shock protein 70, and serotonin: novel targets of beta-hydroxybutyrate in HFFD/LPS-induced sporadic Alzheimer’s disease model
Source: Inflammopharmacology. 2025 May 4;33(6):3461–77. doi: 10.1007/s10787-025-01754-6 (PMC12213920; doi:10.1007/s10787-025-01754-6)
Supplement: Supplementary file 1 — Supplementary file1 (PDF 179 kb) [file 10787_2025_1754_MOESM1_ESM.pdf]

Chaperone-Mediated Autophagy, Heat Shock Protein 70, and Serotonin: Novel Targets of Beta-Hydroxybutyrate in HFFD/LPS-Induced Sporadic Alzheimer's Disease Model

Reem A. Mohamed [a*], Dalaal M. Abdallah[b], Hanan S. El-Abhar[b]

[a]Department of Pharmacology and Toxicology, Faculty of Pharmacy, October University for Modern Sciences and Arts, Cairo, Egypt

[b]Department of Pharmacology and Toxicology, Faculty of Pharmacy, Cairo University, Cairo, Egypt

***Corresponding author:** Reem Ali Mohamed, Department of Pharmacology and Toxicology, Faculty of Pharmacy, October University for Modern Science and Arts (MSA), 26 July Mehwar Road intersection with Wahat Road, 6th of October City. Egypt, 12566

E-mail: ralia@msa.edu.eg, Ralia2025n@gmail.com Tel. number: 002-01224611087

Inflammopharmacology Journal

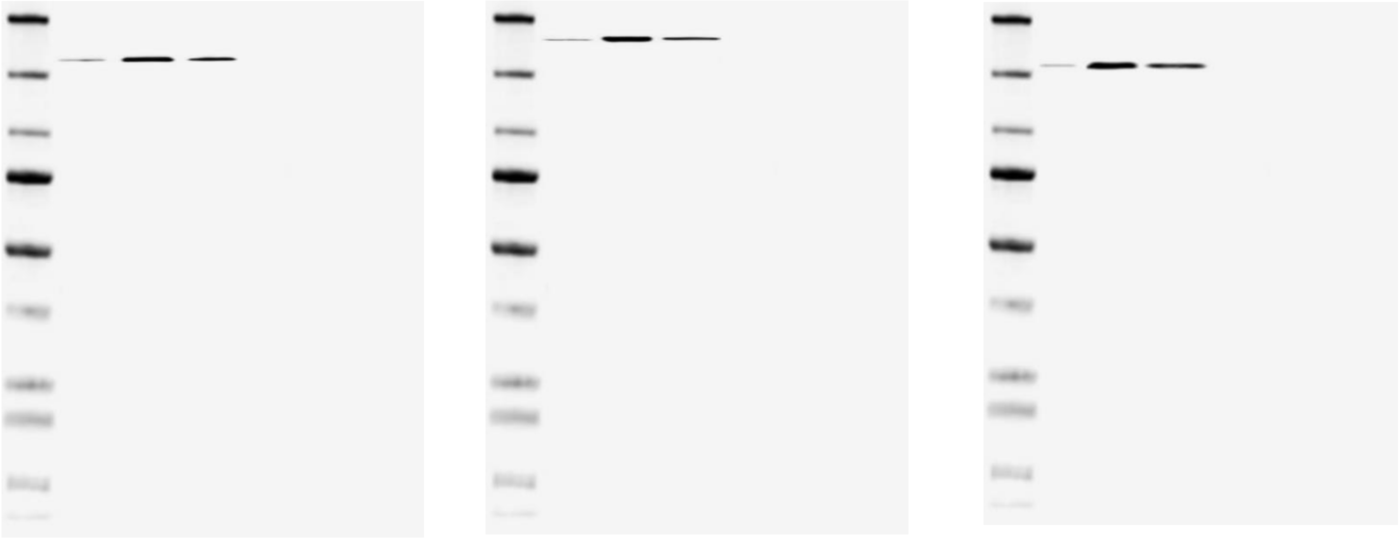

Figure S1: Full-length image of the blotting for NLRP3 Fig 4 (a) (3 repeats)

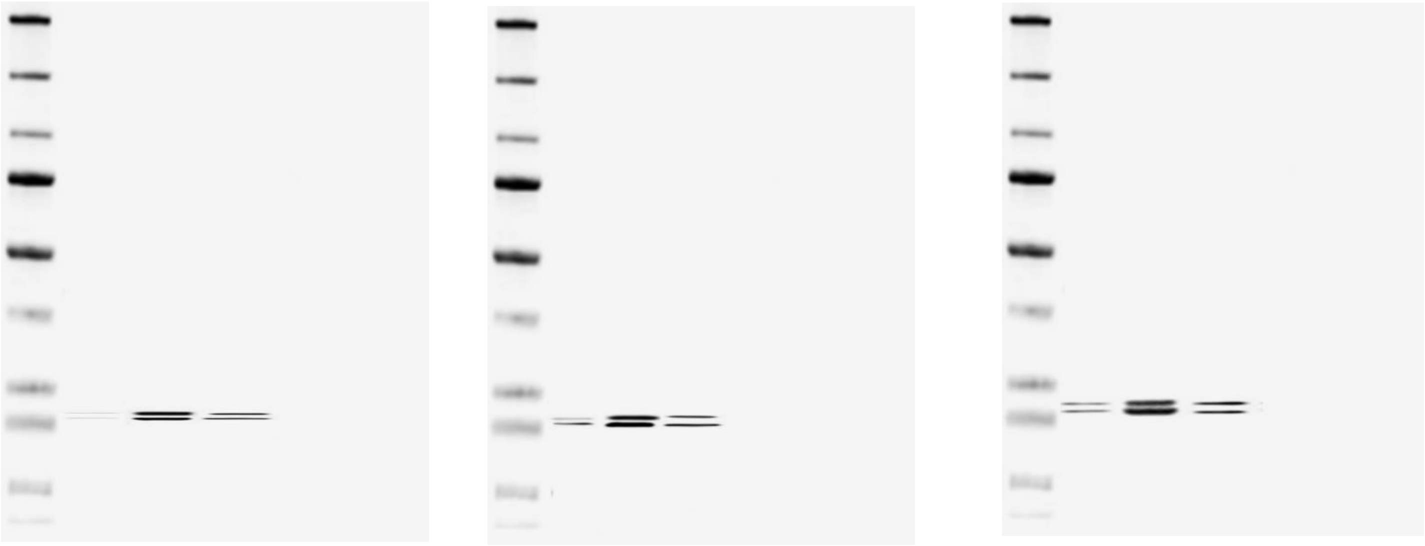

Figure S2: Full-length image of the blotting for Cleaved caspase-1 Fig 4 (b) (3 repeats)

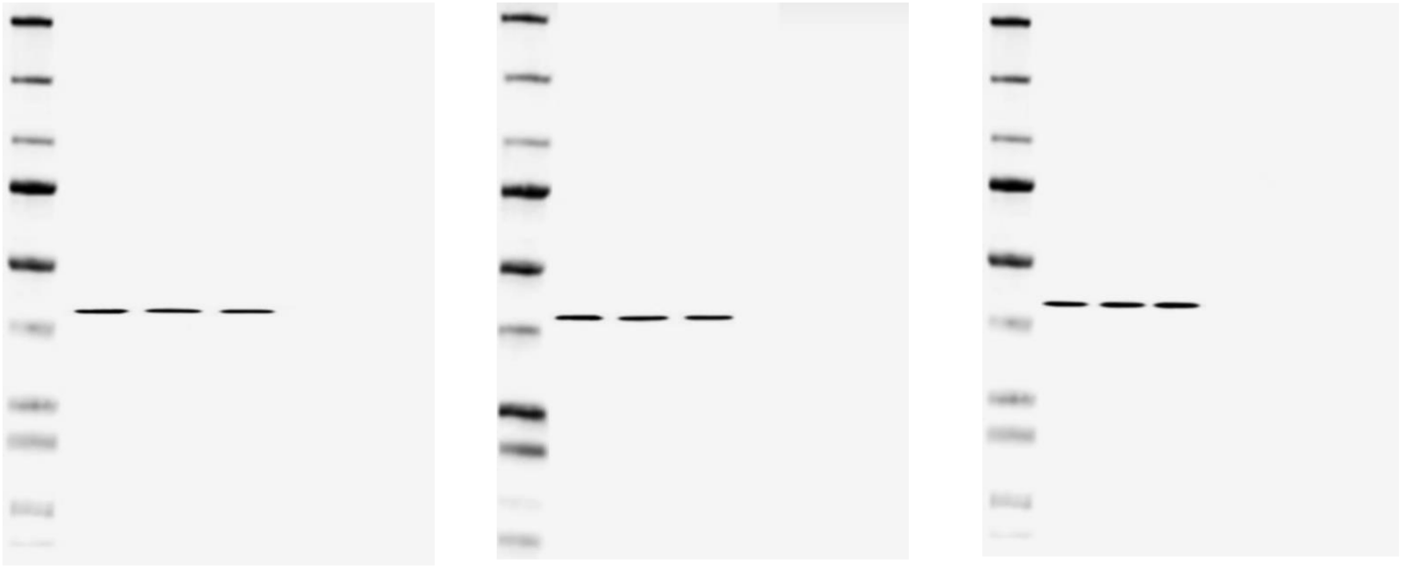

Figure S3: Full-length image of the blotting for β-actin-Figure 4 (a and b) (3 repeats)
